# Palmitoylation: A New Regulatory Role in Meiosis

**DOI:** 10.1371/journal.pbio.1001598

**Published:** 2013-07-02

**Authors:** Caitlin Sedwick

**Affiliations:** Freelance Science Writer, San Diego, California, United States of America

The addition of lipid moieties to a protein can alter its localization, activity, and ability to associate with other proteins within the cell. There are several types of lipid modifications cells can employ on their proteins, but only one of these, palmitoylation (the addition of a palmitate chain), is reversible. This fact has led scientists to conjecture that cells may be able to fine tune the palmitoylation of their proteins in the same way that they can protein phosphorylation. If this were so, it would mean that cells have an entire regulatory scheme that is currently poorly understood. But, to what extent such regulation occurs and how it takes place is currently unknown, so Mingzi Zhang, Howard Hang, and colleagues decided to take a closer look at protein palmitoylation in their article published in *PLOS Biology*.

**Figure:**
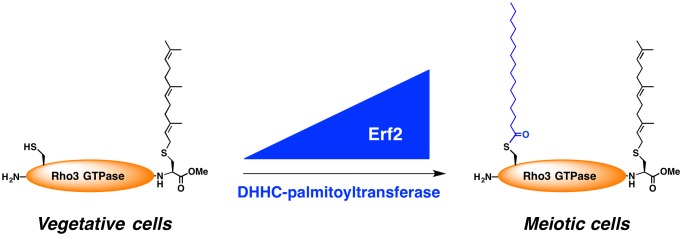
The addition of palmitic acid onto a small GTPase (Rho3) is tuned by precise levels of the Erf2 DHHC-palmitoyltransferase, which helps fission yeast cells undergo sexual differentiation (meiosis).

Zhang et al. studied protein palmitoylation in the fission yeast, *Schizosaccharomyces pombe*. Fission yeast cells possess three chromosomes and normally undergo what's called vegetative growth. In vegetative growth, yeast cells undergo mitosis; each chromosome is copied once and the genetic material is then divvied up evenly between two daughter cells. But, when starved of nutrients, fission yeast can enter a different growth program wherein two cells combine to generate “diploid" cells with six chromosomes. After copying their chromosomes once, diploids undergo two rounds of meiotic divisions to generate four daughter cells—each with three chromosomes but a different genetic makeup and different shot at survival than either parent cell. Several proteins must cooperate to trigger and control this alternative growth program, and the authors wondered whether protein palmitoylation patterns might change during meiotic division.

To detect palmitoylated proteins, the group employed a fatty acid reporter that can be installed onto target proteins by endogenous enzymes in living cells and sequentially visualized using bioorthogonal chemical methods. By applying this technology to cultures of a mutant yeast strain in which meiotic division can be synchronously induced in all cells, the researchers demonstrated that different proteins are palmitoylated during meiosis than in vegetative growth.

Protein palmitoylation is carried out by a family of enzymes called palmitoyltransferases, and Zhang et al. observed that expression of one particular palmitoyltransferase, Erf2, increases during meiosis. So, they postulated that Erf2 might be involved in creating the meiotic palmitoylation pattern. Indeed, they observed that protein palmitoylation is drastically altered and meiosis is delayed in meiotic cells lacking the genes for either Erf2 or its enzymatic cofactor Erf4. This was not the case in cells lacking other palmitoyltransferases the group tested.

To find out which proteins Erf2 modifies during meiosis, the authors immunoprecipitated fatty acid reporter-tagged proteins and identified them by mass spectrometry. This analysis turned up three proteins that are prominently palmitoylated in meiotic cells: Ras1, Isp3, and Rho3. The researchers decided to focus on Ras1 and Rho3, as these small GTPases are often important for cell signaling.

Ras1 and Rho3 are expressed in both vegetative and meiotic cells. But, Zhang and colleagues showed that Ras1 is palmitoylated to the same extent in meiotic cells as it is in vegetative cells, whereas Rho3 is only palmitoylated in meiotic cells by Erf2. This suggests that acute palmitoylation of Rho3 but not of Ras1 is important for meiosis.

Zhang et al. then demonstrate that simply changing the expression levels of the palmitoyltransferase Erf2 is sufficient to increase the palmitoylation levels and subsequent behavior of specific substrates such Rho3. This in turn can affect major cell transitions such as meiosis. To what extent other layers of regulation—such as changes to palmitoyltransferase localization or activity—affect cellular palmitoylation patterns remains to be seen, but this work gives us a glimpse of the possibilities.

**Zhang MM, Wu P-YJ, Kelly FD, Nurse P, Hang HC (2013) Quantitative Control of Protein** ***S*** **-Palmitoylation Regulates Meiotic Entry in Fission Yeast. doi:10.1371/journal.pbio.1001597**

